# The role of ferroptosis in osteoporosis: from pathogenic mechanisms to natural product-driven therapeutic innovations

**DOI:** 10.3389/fmed.2025.1713327

**Published:** 2026-01-02

**Authors:** Yilin Zhu, Xing Wu, Xiao Peng, Hanwei He, Zheng Hu, Liangyi Xiao

**Affiliations:** Changsha Hospital of Traditional Chinese Medicine (Changsha No. 8 Hospital), Changsha City, Hunan Province, China

**Keywords:** ferroptosis, lipid peroxidation, natural active ingredients, osteoporosis, traditional Chinese medicine

## Abstract

Osteoporosis is, a common bone disease, and it has become a prominent health problem of the elderly in modern society. As a new type of cell death, ferroptosis plays an important role in the occurrence and development of osteoporosis. Therefore, regulation of iron metabolism is helpful for the treatment of osteoporosis. This article not only reviews the molecular mechanism of ferroptosis in osteoporosis, but also focuses on the three-dimensional regulatory network of iron metabolism disorder, lipid peroxidation, and bone homeostasis imbalance. Natural active ingredients with potential inhibiting ferroptosis, including traditional Chinese medicine, and their corresponding targets, are also evaluated from the perspective of natural product chemistry and molecular pharmacology. Finally, the research and development prospects of natural ingredient delivery systems in the treatment of osteoporosis are discussed. Advances in these therapeutic strategies provide new opportunities to address the challenges in the management of osteoporosis and may improve the quality of life of elderly patients. This article comprehensively reviews the related studies of ferroptosis and osteoporosis, providing a valuable reference for research and clinical practice in this field, and contributing to the further development of osteoporosis treatment research.

## 1 Introduction

Osteoporosis is a common skeletal condition marked by a decrease in bone mass and the degradation of the microstructure of bone tissue, resulting in increased fragility of bones and an elevated likelihood of fractures (1). The primary clinical manifestations include bone pain, fatigue, spinal deformity, and fractures (2). With the intensification of population aging, the incidence of fractures and complications associated with osteoporosis is rising annually, significantly impacting public health. Consequently, osteoporosis has emerged as a prominent health concern in modern society. The National Osteoporosis Foundation approximates that 10.2 million individuals in the United States suffer from osteoporosis, while an extra 43.4 million exhibit low bone mass. Additionally, forecasts indicate that by 2030, the total number of adults dealing with osteoporosis and low bone mass is expected to increase to 71 million (3). According to the latest statistics from China, the incidence of osteoporosis among individuals aged 50 and above is 19.2%, with rates of 6.0% in men and 32.1% in women (4). In European countries, the prevalence among men over 50 is 7.2% (Luxembourg), and among women, it is 23.5% (France) (5).

In recent times, ferroptosis, an innovative type of cellular demise, has attracted considerable interest within the domain of bone metabolism. A growing number of research suggests a strong connection between ferroptosis and osteoporosis. The regulation of iron metabolism plays a crucial role in upholding the body’s iron homeostasis, involving iron uptake, storage, transport, and utilization. As a key component of hemoglobin, cytochromes, and iron–sulfur clusters, iron is essential for various biological processes, including the transport of oxygen, energy metabolism, and DNA synthesis, among others. In contrast to classical forms of cell death such as necrosis and apoptosis, ferroptosis is distinguished by its lack of cell membrane rupture or swelling, and it does not cause cellular shrinkage, pyknosis, nuclear fragmentation, or the formation of apoptotic bodies. The defining features of ferroptosis include a reduction in size and a decrease in mitochondria, potential elimination of cristae, increased membrane density, and membrane rupture, along with blebbing, while exhibiting minimal changes in nuclear morphology (6). The mechanism of ferroptosis is primarily associated with disorders in iron metabolism, imbalances in the amino acid antioxidant system, and the accumulation of lipid peroxides. The buildup of lipid peroxides plays a key role in ferroptosis, while glutathione peroxidase 4 (GPX4), an essential antioxidant enzyme, is capable of blocking, thus inhibiting ferroptosis (7). Moreover, an excess of iron plays a crucial role in ferroptosis, since iron ions produce a considerable quantity of reactive oxygen species via the Fenton reaction, which in turn enhances lipid peroxidation and triggers ferroptosis (8).

This review intends to explore the molecular mechanisms underlying ferroptosis in the development and progression of osteoporosis, focusing on elucidating the three-dimensional regulatory network involving iron metabolism disorders, lipid peroxidation, and imbalances in bone homeostasis. At the same time, it assesses natural active compounds with potential anti-ferroptosis effects and their respective targets, drawing from the fields of natural product chemistry and molecular pharmacology.

Finally, it explores the research and development prospects of natural component delivery systems (such as exosomes) based on ferroptosis regulation, aiming to provide a theoretical basis and new directions for drug development in the precise prevention and treatment of osteoporosis.

## 2 The mechanism between osteoporosis and ferroptosis

### 2.1 Ferroptosis affects bone metabolism

#### 2.1.1 Iron metabolism disorder

The communication between osteoblasts and osteoclasts is essential for the fine regulation of bone remodeling in the process of maintaining bone homeostasis (9). During this process, osteoclasts attach to the old bone and dissolve minerals and digest bone matrix by releasing proteases and acidic products. Osteoblasts, on the other hand, guide the deposition of extracellular matrix and promote bone formation by secreting alkaline phosphatase, osteopontin and osteocalcin. An elevation in osteoclast activity or a reduction in osteoblast activity caused by any reason will disrupt the balance and lead to the occurrence of osteoporosis. Iron, an important trace element in the human body, plays a vital role in the regulation of bone homeostasis. Whether iron overload or deficiency, it will affect the differentiation and activity of osteoclasts and osteoblasts, thereby disrupting the bone homeostasis and accelerating bone loss. For instance, iron deficiency can directly or indirectly regulate the activity and function of osteoblasts and osteoclasts by inducing hypoxia and disorder of vitamin D metabolism, ultimately destructing bone homeostasis (10). Furthermore, extremely low levels of iron concentration inhibit the activity of osteoblasts, affect their functions, and subsequently lead to osteoporosis (11). When the iron concentration is excessive, osteoblasts are inibited, while osteoclasts maintain mitochondrial function through excessive iron to complete the process of bone resorption, thereby disrupting bone homeostasis (12).

Abnormal iron metabolism resulting in iron overload is a key feature of ferroptosis. This surplus of free iron ions produces an overabundance of reactive oxygen species (ROS) through the Fenton reaction and enhances the activity of lipoxygenase. Lipoxygenase can cause lipid peroxidation of unsaturated fatty acids in the cell membrane, thereby inhibiting cell activity (13, 14). Studies have shown that iron overload reduces the viability of mouse embryonic osteoblast precursor cells (MC3T3-E1) and induces cell apoptosis (15). And this leads to a proliferation defect and imbalance in osteogenic/adipogenic differentiation of bone marrow mesenchyml stem cell (BMSCs) (16). Iron overload also promotes oxidative stress, leading to trabecular bone damage and bone homeostasis imbalance, which in turn causes bone loss in mice (17). Furthermore, excessive iron can also increase the ratio of RANKL/osteoprotegerin (OPG) in bone cells, ultimately enhancing the differentiation and bone-resorbing function of osteoclasts, and inducing the occurrence of osteoporosis (18).

Mitochondria serve as crucial storage locations for iron ions in cells, and alterations in their ultrastructure are key features of ferroptosis. Additionally, disturbances in mitochondrial iron metabolism are strongly associated with ferroptosis (19). For example, irregular expression or dysfunction of mitochondrial ferritin may result in mitochondrial iron ion overload, thereby promoting lipid peroxidation and ferroptosis.

Moreover, during ferroptosis, the generation of ROS can exacerbate lipid peroxidation, thereby establishing a harmful cycle that facilitates the onset of ferroptosis (20). In conclusion, iron metabolism is closely related to the bone microenvironment, and ferroptosis may play an important role in the pathogenesis of osteoporosis.

#### 2.1.2 Lipid peroxidation

Lipid peroxidation reactions exacerbate the process of ferroptosis. Free polyunsaturated fatty acids (PUFAs) serve as crucial substrates for lipid oxidation. Their binding to the phospholipid bilayer leads to excessive oxidation, which damages both the mitochondrial membrane and the cell membrane, subsequently triggering ferroptosis (21). Molecules such as lysophosphatidylcholine acyltransferase-3 (LPCAT3) and long-chain acyl-CoA synthetase 4 (ACSL4) also influence the lipid peroxidation process of membrane PUFAs and aggravating ferroptosis (22). By modulating signaling pathways, reducing the levels of ROS, and enhancing the activity of GPX4, the toxicity of lipid peroxides can be mitigated, thereby inhibiting osteoblast ferroptosis (23, 24). Therefore, preventing the buildup of lipid peroxides appears to be a promising approach for addressing ferroptosis.

#### 2.1.3 Imbalance of the amino acid antioxidant system

The disruption of the amino acid antioxidant system plays a crucial role in ferroptosis. System Xc- functions as an anti-transporter that is extensively found within the phospholipid bilayer, serving as an essential component of the cellular antioxidant framework (25). It can transport extracellular cystine into the cell and serve as a raw material for cysteine production, further synthesizing glutathione (GSH) (26). GPX4 is an important component of the human antioxidant system. Under the action of GPX4, GSH maintains a dynamic equilibrium with oxidized glutathione (GSSH). As an auxiliary factor of GPX4, GSH can reduce the toxicity of lipid peroxides and protect the biological membrane system from damage caused by ferroptosis. Studies have shown that activating transcription factor 3 (ATF3) can mediate ferroptosis of osteoblasts in a high-glucose environment by inhibiting the activity of System Xc-, thereby inducing osteoporosis (27), and more studies have targeted GPX4 to inhibit ferroptosis, with the aim of treating osteoporosis (23, 24). Although GPX4 is the primary mechanism for inhibiting iron poisoning, studies have shown that cells deficient in GPX4 can still survive in the activated state of ferroptosis. Based on this observation, a previously unknown iron death suppressor protein 1 (FSP1) was discovered. Further research confirmed that FSP1 inhibits ferroptosis through NAD(P)H-dependent coenzyme Q10. The protective role of FSP1 is realized through its ability to catalyze the ongoing regeneration of CoQ10 and enhancing the capacity to capture free radicals, thereby inhibiting ferroptosis. Notably, FSP1 demonstrates a protective effect against ferroptosis that is triggered by the deficiency of GPX4 (28, 29).

### 2.2 Bone-related cells and ferroptosis

#### 2.2.1 Osteoblasts and ferroptosis

Osteoblasts are pivotal cells in bone tissue formation, originating from BMSCs. They not only synthesize and secrete the organic components of the bone matrix but also release matrix vesicles, participating in matrix mineralization. In terms of bone physiology, iron deficiency was previously thought to adversely affect bone homeostasis (30). However, studies indicate that low levels of iron exhibit a biphasic influence on osteoblasts; mild deficiency in iron enhances the activity of osteoblasts, while a severe deficiency hampers their activity (11). Moreover, increased iron levels due to ferroptosis have also been reported as one of the factors inhibiting bone formation.

**Figure 1:**
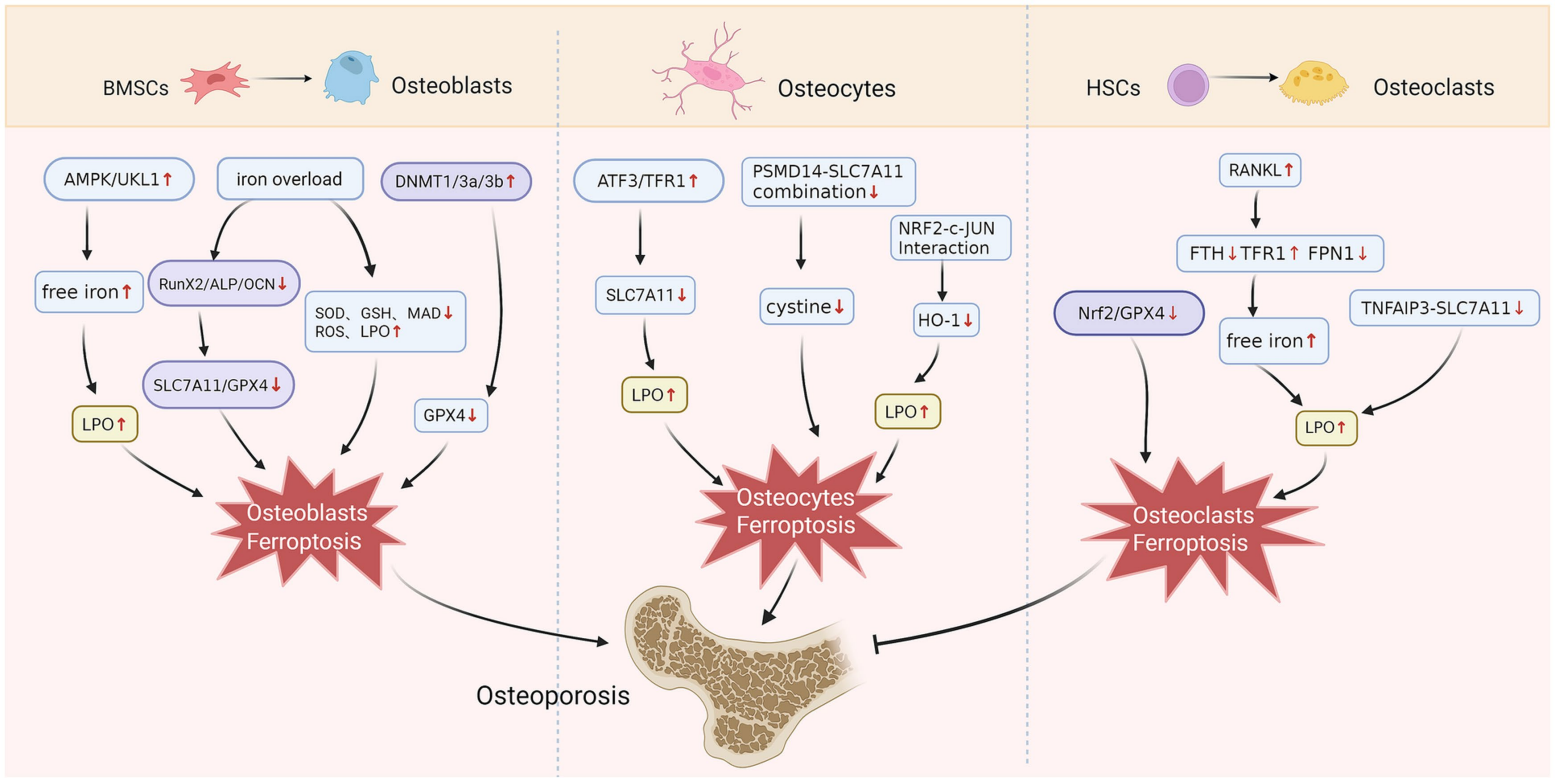
The effect of ferroptosis on bone-related cells. Osteoblasts originate from BMSCs. Iron overload exacerbates lipid peroxidation by modulating the RUNX2/ALP/OCN axis or affecting ferroptosis-related proteins, ultimately inducing osteoblast ferroptosis. AMPK/UKL1 promotes osteoblast ferroptosis by affecting free iron levels, while DNMT1/3a/3b inhibits GPX4 expression. Activating ATF3/TFR1 to suppress SLC7A11, or inhibiting HO-1 via NRF2-c-JUN interaction, can exacerbate lipid peroxidation and promote osteocytotoxic ferroptosis. The reduced binding of PSMD14-SLC7A11 inhibits cystine expression, inducing osteoclast ferroptosis and promoting osteoporosis. Osteoclasts derive from the HSCs. RANKL induces osteoclast ferroptosis via the iron starvation response mechanism, thereby inhibiting osteoporosis. Inhibition of MZF1 by promoting the Nrf2/GPX4 axis can suppress osteoclast ferroptosis and promote osteoporosis.

Studies demonstrate that iron overload inhibits bone formation and promotes osteoporosis. The mechanism involves iron overload suppressing the expression of bone formation-related transcription factors, including runt-related transcription factor 2 (RunX2), osteocalcin (OCN), and alkaline phosphatase (ALP), which subsequently reduces SLC7A11/GPX4 levels and ultimately induces ferroptosis (31). Furthermore, iron overload reduces superoxide dismutase (SOD), GSH, and mitochondrial adenosine dinucleotide (MAD) levels, increases ROS production, exacerbates lipid peroxidation, and promotes osteoblast ferroptosis, thereby impairing osteoblast function. Additionally, iron overload induces mitochondrial ultrastructural alterations in MC3T3-E1 cells, including generalized mitochondrial enucleation, increased mitochondrial membrane density, and reduced cristae number, with some cases showing complete disappearance (32). Recent studies have reported that DNMT abnormalities induce epigenetic suppression of GPX4, which subsequently triggers osteoblast ferroptosis, which is a key mechanism underlying osteoporosis (33). Furthermore, activation of the AMPK/ULK1 signaling pathway promotes selective ferroptosis of ferritin, leading to free iron release and lipid peroxidation accumulation, ultimately causing osteoblast ferroptosis (34). In summary, osteoblast ferroptosis may be one of the mechanisms that affect bone formation and osteoporosis (Figure 1).

#### 2.2.2 Osteoclasts and ferroptosis

Osteoclasts, derived from the monocyte–macrophage lineage within the hematopoietic stem cells (HSCs), play a vital role in the process of bone resorption in the human body. Osteoclasts primarily acquire iron through transferrin receptor 1 (TFR1)-mediated iron uptake, a mechanism that facilitates osteoclast differentiation and bone resorption (35). The results showed that RANKL induced osteoclast ferroptosis under normal oxygen condition, and the key mechanism was iron starvation response, which was manifested as decreased ferritin heavy chain (FTH), ferroportin (FPN) and increased TFR1. This process led to the increase of free iron ion concentration, which promoted lipid peroxidation and finally induced osteoclast ferroptosis (36). The downregulation of myeloid zinc finger 1 (MZF1) expression, which activates the Nrf2/GPX4 pathway and inhibits rankl-induced ferroptosis in osteoclasts during early cell differentiation, may be a key mechanism underlying ovarian removal (OVX) mice models of postmenopausal osteoporosis (37). Recent studies have reported that the natural diphenylstilbene compound Gnetol (GT) can effectively induce osteoclast ferroptosis via the TNFAIP3-SLC7A11 axis, providing a novel therapeutic approach for osteoporosis (38) (Figure 1).

#### 2.2.3 Osteocytes and ferroptosis

Osteocytes, which are the predominant cell type in bone tissue, have the ability to detect mechanical stress and manage both the formation and breakdown of bone. Consequently, they are regarded as the main regulators of bone activity. Recent research has progressively acknowledged the essential function of osteocytes in the processes of bone remodeling and maintaining homeostasis (39). The study revealed that high expression of ATF3 in senescent osteocytes promotes iron uptake by upregulating TFR1, while simultaneously inhibiting cystine uptake mediated by SLC7A11. This leads to iron overload and lipid peroxidation, ultimately resulting in ferroptosis (40). Furthermore, glucocorticoids directly inhibit the binding of the deubiquitinating enzyme PSMD14 to SLC7A11, thereby promoting the ubiquitination and subsequent proteasomal degradation of SLC7A11. This significantly reduces cystine uptake, leading to osteoclast apoptosis and ultimately accelerating the development of osteoporosis (41). Furthermore, studies on diabetic osteoporosis (DOP) mouse models demonstrate that heme oxygenase-1 (HO-1) is significantly upregulated in ferroptotic osteocytes. HO-1’s expression is regulated by the interaction between its promoter activity and the upstream transcription factors NRF2 and c-JUN, which plays a pivotal role in DOP-induced ferroptosis of osteocytes (42). Therefore, osteocyte ferroptosis may accelerate the process of osteoporosis by interfering with the balance of bone formation and bone resorption (Figure 1).

### 2.3 The signaling pathway mechanisms of ferroptosis in regulating bone metabolism

Ferroptosis significantly impacts the metabolic balance of bone through various mechanisms and signaling pathways, thereby contributing to the promotion of osteoporosis. The primary mechanisms and pathways involved are as follows.

#### 2.3.1 Antioxidant stress pathway

The Nrf2/HO-1 signaling pathway plays a vital role in regulating iron balance and demonstrates effects that are anti-resorptive to bone, as well as protective against oxidative stress, inflammation, and apoptosis. By activating the Nrf2/HO-1 pathway, the levels of ROS are reduced, while the levels of solute carrier family 7 member 11 (SLC7A11) are increased, enhancing GPX4 activity. This cascade ultimately mitigates the toxicity of lipid peroxides and inhibits ferroptosis in osteoblasts (23, 24). Additionally, a study has shown that dexamethasone (DEX) induces glucocorticoid-induced osteonecrosis and MC3T3-E1 cells ferroptosis by regulating P53/SLC7A11/GPX4 axis (43), indicating that this pathway may be a potential strategy for treating osteoporosis. Moreover, the ASK1- p38 signaling pathway plays a crucial role in regulating the survival, differentiation, and function of osteoblasts under the influence of stress signals (44).

#### 2.3.2 Iron homeostasis regulatory pathway

The iron regulatory pathway is crucial for the induction of osteoporosis through ferroptosis. The research indicates that the absence of NOL1/NOP2/Sun domain family, member 5 (NSUN5) leads to a reduction in the levels of 5-methylcytosine in ferritin heavy chain 1(FTH1)/ferritin light chain (FTL) RNA, which results in increased iron concentration in BMSCs, downregulation of GPX4, and accumulation of ROS and lipid peroxidation products (45). Therefore, modulating the NSUN5-FTH1/FTL pathway presents a potential strategy to inhibit ferroptosis and enhance the survival rate of BMSCs (45).

#### 2.3.3 Cellular metabolism and survival regulation pathways

The PI3K/AKT/mTOR pathway, as a classic pro-survival pathway, is involved in the maintenance of bone homeostasis. Studies have found that high-dose DEX induces ferroptosis in BMSCs through the PI3K/AKT/mTOR signaling pathway (46). Melatonin notably reduces glucocorticoid-triggered ferroptosis in BMSCs and prevents ferroptosis through the stimulation of the PI3K/AKT/mTOR signaling pathway, thereby preventing the onset of steroid-induced osteoporosis (SIOP) (43).

#### 2.3.4 Bone remodeling balance regulatory pathway

The RANKL/OPG pathway serves a crucial regulatory role in the differentiation and activation of osteoclasts, with its ratio being a significant determinant of bone integrity and mass (47). Elevated iron levels lead to an increased RANKL/OPG ratio, which enhances osteoclast differentiation and bone resorption (48). Conversely, effectively reducing the RANKL/OPG ratio, increasing the expression of GPX4, and inhibiting the ferroptosis pathway can thereby treat osteoporosis (49, 50).

**Figure 2:**
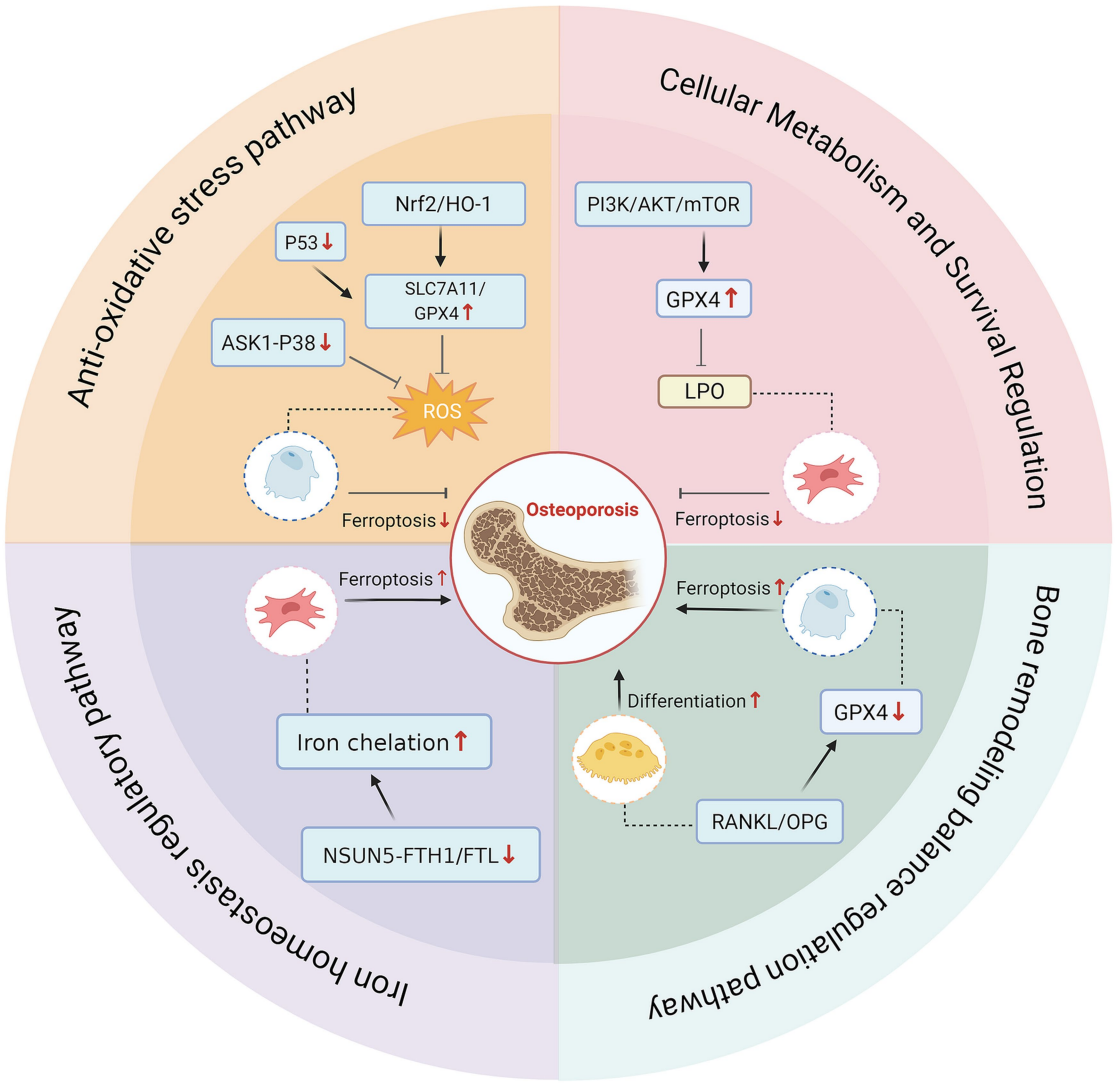
The signaling pathway mechanisms of ferroptosis in regulating bone metabolism. Key signaling pathways regulating ferroptosis in bone cells and their significant roles in bone homeostasis. The Nrf2/HO-1 pathway effectively inhibits osteoblast ferroptosis by reducing ROS, upregulating SLC7A11 gene expression, and enhancing GPX4 activity. The P53/SLC7A11/GPX4 and ASK1-P38 pathways suppress ferroptosis by inhibiting ROS. NSUN5 gene deletion induces abnormal iron storage through reduced FTH1/FTL RNA methylation, leading to iron overload and ferroptosis that disrupt iron homeostasis. Meanwhile, the PI3K/AKT/mTOR pathway promotes cell survival and regulates lipid peroxidation to inhibit BMSCs ferroptosis. Additionally, the RANKL/OPG pathway induces osteoclast differentiation while blocking osteoblast ferroptosis through GPX4 expression suppression.

In conclusion, bone metabolism is affected by ferroptosis via multiple pathways, playing a role in the progression of osteoporosis. Targeting these signaling pathways presents potential strategies for the treatment of osteoporosis. Future research should further elucidate the interactions among different pathways and investigate drug delivery systems that can specifically modulate ferroptosis (Figure 2).

#### 2.3.5 Emerging regulatory factors and interaction networks

In recent years, research into the mechanisms of ferroptosis across various fields has led to the discovery of numerous new targets and pathways that have garnered significant attention. Sirtuin 1 (SIRT1) plays a critical regulatory role in ferroptosis. The downregulation of SIRT1 diminishes the inhibitory effect of vitamin K-2 (VK2) on ferroptosis in BMSCs exposed to high glucose, consequently contributing to bone loss (51). Recent research indicates that ELAV-like RNA binding protein 1 (ELAVL1) inhibits the translation of SIRT1, disrupts osteoblast differentiation mediated by ferroptosis, and thereby impedes the progression of osteoporosis (52). Furthermore, some studies have proposed a novel mechanism involving the NRF2-iron-ornithine metabolic axis in osteoclasts. In this study, the glycine uptake inhibitor bitopertin was employed to diminish the binding of kelch-like ECH-associated protein 1(Keap1)-Nrf2, resulting in a reduction of Nrf2 protein degradation. Nrf2 transcriptionally activates the gene encoding the iron transporter Slc40a1, thereby decreasing the iron content within osteoclasts. Additionally, the lack of Nrf2 or the addition of iron leads to an increase in the activity of the ornithine metabolism enzyme Odc1, which subsequently reduces ornithine levels and accelerates osteoclast differentiation (53). However, it is important to note that this study was conducted solely in mice, and no clinical trials have been performed to assess its feasibility and safety in humans. Further investigations have revealed that ferroptosis represents a significant mode of bone cell death in cortical bone during the aging process. Moreover, single-cell transcriptome analysis has determined that the ATF3 is a key driver of bone cell ferroptosis. The elevated level of ATF3 in aging bone cells promotes iron absorption through the upregulation of TFR1, while concurrently suppressing cystine uptake facilitated by SLC7A11. The described process causes an accumulation of iron and increases lipid peroxidation, which eventually results in ferroptosis and worsens the reduction of cortical bone mass (40). The results from this research offer encouraging approaches for both the prevention and management of osteoporosis and fractures.

### 2.4 The relationship between ferroptosis and different clinical subtypes of osteoporosis

The clinical subtypes of osteoporosis primarily include postmenopausal osteoporosis, diabetic osteoporosis, glucocorticoid-induced osteoporosis, and thalassemia-related osteoporosis. Although the core driving factors of these various types of osteoporosis differ, existing studies indicate aclose relationship with ferroptosis. However, the triggers that initiate ferroptosis, along with the associated mechanisms and cellular targets, exhibit distinct characteristics (54, 55) (Table 1). Furthermore, we have found that diabetes-induced osteoporosis, post-menopausal osteoporosis, and senile osteoporosis are associated with certain endocrine levels. This indicates that they may have different treatment strategies. For instance, estrogen replacement therapy is used to treat post-menopausal osteoporosis (56, 57). Regulating insulin signaling in osteoblasts promotes the activity of osteocalcin in patients with diabetic osteoporosis (58), and supplementing vitamin D and using parathyroid hormone analogs can treat senile osteoporosis (59). However, at present, there is still a lack of direct evidence to prove that regulating the endocrine level can treat osteoporosis by correcting ferroptosis.

**Table 1:** Ferroptosis and different types of osteoporosis.

OP subtype	Clinical characteristics	Core trigger	Key ferroptotic pathway	Distinct cell target	References
Postmenopausal	Postmenopausal female patients; menopausal symptoms like flushing	The estrogen level in postmenopausal women decreases	Nrf2/Dnmt3a/RANKL	Osteocyte; osteoclast	(105)
Glucocorticoid-induced	Patients who have used glucocorticoids for a long time; a higher risk of fractures	Extensive use of glucocorticoids	PI3K/AKT/mTOR; SLC7A11/GPX4	BMSC osteocyte	(41, 46)
Diabetic	Long-term diabetic patients; diabetes often leads to complications such as sarcopenia	The microenvironment of diabetes	METTL3/ASK1-p38; AMPK/SIRT1	Osteoblast; BMSC	(44, 51)
Thalassemia-related	Patients with thalassemia who have undergone repeated blood transfusions are mostly teenagers and young adults, accompanied by bone deformities, anemia, delayed growth and development, etc.	Repeated blood transfusion treatments lead to iron overload	transient receptor potential vanilloid type 1 (TRPV1) channels	Osteoclast	(106)

## 3 Targeting ferroptosis anti-osteoporosis drugs

The primary features of ferroptosis include an excess of iron and the buildup of ROS, with lipid peroxidation being its key mechanism (60). Therefore, inhibiting lipid peroxidation, regulating iron metabolism, and counteracting oxidative stress are crucial strategies for treating ferroptosis and osteoporosis. The primary features of ferroptosis include an excess of iron and the buildup of reactive oxygen species, with lipid peroxidation serving as its essential mechanism.

### 3.1 Regulation of iron metabolism

Iron chelators demonstrate significant clinical efficacy in patients with osteoporosis resulting from iron overload, primarily by reducing intracellular free iron levels. Currently, the iron chelators commonly utilized in clinical practice include deferoxamine, deferiprone, and deferasirox (61). Desferrioxamine methylsulfonate has been demonstrated to effectively increase bone density in postmenopausal patients with osteoporosis (62). This compound not only positively influences the maturation and function of osteoblasts (12), but also reduces the generation of osteoclasts and inhibits the enzymatic activity of mitochondrial complexes along with the expression of their subunits (63, 64). As a bone-forming angiogenic agent, desferroxamine enhances vascularization at the metaphysis and promotes osteoblast activity, thereby preventing and treating bone loss in estrogen-deficient mice (65). Furthermore, desferrioxamine exhibits a protective effect against osteogenesis inhibition induced by iron overload in zebrafish models, restoring mineralization ability by inhibiting ferroptosis (66). Similarly, deferiprone reduces iron ion concentration in osteoblasts, increases the activity of ALP and demonstrates a stronger effect than deferoxamine (67). Interestingly, other research suggests that deferoxamine can prevent ferroptosis and maintain osteoblast differentiation, irrespective of iron overload clearance (68). Deferasirox is widely utilized for the management of chronic iron overload resulting from frequent blood transfusions in pediatric patients with β-thalassemia. Furthermore, in adult patients with transfusion-dependent β-thalassemia who receiving dilazep therapy, the incidence of osteoporosis was markedly diminished (69, 70). Thalassemia patients receiving long-term deferasirox treatment, regardless of their use of bisphosphonate therapy, hormone replacement therapy, or supplements of calcium and vitamin D, observed a noteworthy rise in average lumbar spine bone mineral density (71). Additionally, there was a significant decrease in the number of patients identified with lumbar spine osteoporosis (71). Deferasirox showed therapeutic effects in reducing bone loss in postmenopausal mice with iron accumulation. The mechanism might be through lowering serum ferritin levels, reducing iron overload, inhibiting ferroptosis, thereby increasing bone trabeculae and enhancing bone density (72). *In vitro* investigations have validated that deferasirox can inhibit abnormal bone metabolism induced by iron overload, with its mechanism potentially linked to the suppression of NF-κB pathway activity, thereby impeding the differentiation of mouse monocyte RAW264.7 cells into osteoclasts (73).

In conclusion, regulating iron metabolism is a crucial approach to inhibiting ferroptosis. Commonly used iron chelating agents in clinical practice may treat osteoporosis by reducing iron overload and inhibiting ferroptosis, presenting a potential strategy for managing this disease.

### 3.2 Antioxidant stress and inhibition of lipid peroxidation

Vitamins are natural antioxidants. For instance, nutrients such as vitamin D, vitamin K, and calcium play an important role in the maintenance of healthy bones. Vitamins may prevent and treat osteoporosis by mechanisms such as antioxidative stress and inhibition of lipid peroxidation, thereby blocking ferroptosis (Table 2).

**Table 2:** Vitamin-regulated ferroptosis in the treatment of osteoporosis.

Ingredient	Source	Mechanism	Effect	References
Vitamin K2	Fermented foods (such as natto)	Activation of the AMPK/SIRT1 pathway inhibits high glucose-induced bone loss and ferroptosis	Improve bone microstructure in diabetes models and reduce the lipid peroxidation marker malondialdehyde (MDA)	(51)
Vitamin D	Sunlight exposure, vitamin D-rich foods (such as fish, milk)	Activate the Nrf2/GPX4 signaling pathway, downregulate lipid peroxidation, and alleviate osteoblast ferroptosis	Downregulation of d-galactose (d-gal)-induced osteoblast cellular senescence markers	(24)
Vitamin E	Vegetable oil (such as soybean)	Inhibiting the ferroptosis pathway of BMSCs induced by H_2_O_2_ through the PI3K/AKT/mTOR pathway	Alleviating oxidative stress in BMSCs to promote osteogenic differentiation	(107)

Furthermore, there are some drugs that can treat osteoporosis-related ferroptosis by inhibiting lipid peroxidation and oxidative stress, among other mechanisms. Melatonin, for example, has been demonstrated to prevent osteoblast ferroptosis through the activation of the Nrf2/HO-1 signaling pathway, thereby enhancing bone microstructure in both *in vivo* and *in vitro* studies (23). Zoledronic acid induces osteoclast ferroptosis by triggering the ubiquitination degradation of p53 mediated by F-box protein 9, thereby improving osteoporosis. This is accompanied by an increase in Fe^2+^, ROS and MDA levels, as well as a decrease in GPX4 and GSH levels (74). Interestingly, current research indicates that hypoglycemic drugs also have the potential to treat osteoporosis (75). Experiments *in vitro* demonstrated that metformin inhibits osteoblast ferroptosis induced by high glucose and palmitic acid, significantly enhances the expression of ferroptosis protective proteins (GPX4, FTH1, SLC7A11), and reduces lipid peroxidation products and iron ion levels. *In vivo* studies also suggest that metformin can alleviate bone loss and microstructural deterioration in DOP rats. The mechanism is related to activating the AMPK/Nrf2 pathway, enhancing antioxidant capacity, and inhibiting iron death-related processes (76). Furthermore, rosiglitazone exerts protective effects on the cartilage of mice with osteoarthritis by inhibiting lipid peroxidation and restoring iron homeostasis, and it also shows anti-ferroptosis effects. However, there is still a lack of large-scale clinical studies to support this (77). Rosiglitazone, by inhibiting lipid peroxidation and restoring iron homeostasis, exerts a protective effect on the cartilage of osteoarthritis mice and demonstrates anti-ferroptosis effects (77), indicating that rosiglitazone may treat osteoporosis and other bone joint diseases through this mechanism.

### 3.3 Traditional Chinese medicine (TCM) compound and monomer components

#### 3.3.1 TCM compound prescriptions and single herbs

Numerous individual Chinese herbal medicines and herbal formulas have demonstrated efficacy in the prevention and treatment of osteoporosis, potentially through the inhibition of ferroptosis. Studies indicate that Fructus Ligustri Lucidi significantly inhibits ferroptosis in postmenopausal osteoporotic rats, thereby safeguarding their osteogenic capacity, an effect linked to the activation of the Nrf2/HO-1 signaling pathway (78). Furthermore, Qing e Pill (QEP) mitigates osteoblast apoptosis in primary osteoporosis via the ATM serine/threonine kinase (ATM) and PI3K/AKT pathways, thereby exerting its therapeutic effects on osteoporosis (79). The Eucommia-Dipsacus herb pair enhances serum estradiol levels, as well as GPX4 and FTH1 protein levels in ovariectomized osteoporotic rats, inhibits femoral ferroptosis, and consequently increases femoral bone mineral density, alleviating osteoporosis (80). The latest research also reports that psoraleae fructus combined with walnut kernels improves postmenopausal osteoporosis by inhibiting ferroptosis through the Nrf2/GPX4/SLC7A11 pathway (81). Additionally, Er-Xian decoction inhibits ferroptosis and alleviates osteoporosis caused by ovariectomy by regulating fatty acid metabolism and the IGF1/PI3K/AKT signaling pathway (82). Additionally, Bugushengsui formula regulates oxidative stress and iron accumulation in osteoporosis model rats following bilateral ovariectomy, potentially exerting anti-osteoporotic effects via the cellular ferroptosis pathway (83).

#### 3.3.2 Monomeric components of TCM

The active components of certain traditional Chinese medicines can inhibit ferroptosis through multiple targets and pathways, thereby preventing and treating osteoporosis (Table 3). Mangiferin (C19H18O11) is a flavonoid glucoside commonly isolated from mangoes and is also found in medicinal plants such as anemarrhena asphodeloides, belamcanda chinensis, and gentiana scabra (84–86). Studies have demonstrated that mangiferin promotes bone formation and inhibits ferroptosis in both *in vivo* models (osteoporotic mice, iron-overloaded mice) and *in vitro* models (osteoblast ferroptosis, iron-overloaded osteoblasts) (87). The underlying mechanism involves mangiferin’s direct binding to Keap1 and the subsequent activation of the downstream Nrf2/SLC7A11/GPX4 pathway (87). Asperosaponin VI (AVI), a triterpenoid saponin extracted from dipsacus asper, has the molecular formula C_47_H_76_O_18_. Research indicates that AVI effectively reverses hypermethylation by inhibiting DNMT1/3a, restores GPX4 expression, and mitigates ferroptosis pathology associated with DOP (88). Additionally, recent studies have reported that gstrodin and ginsenoside can improve neurological dysfunction and myocardial ischemia by inhibiting ferroptosis-related pathways (89, 90), suggesting that they may also exert therapeutic effects on osteoporosis through this mechanism, providing a potential therapeutic approach.

**Table 3:** Monomer components of TCM regulating ferroptosis for osteoporosis treatment.

Ingredient	Source	Mechanism	Effect	References
Mangiferin	Anemarrhena asphodeloides, belamcanda chinensis, and gentiana scabra	Bind to Keap1 and subsequently activate the downstream Nrf2/SLC7A11/GPX4 pathway	Promotes bone formation and inhibits ferroptosis inosteoblast	(86, 87)
Asperosaponin VI	Dipsacus asper	Inhibition of DNMT1/3a and restoration of GPX4 expression	Reduced ferroptosis of osteoblasts under DOP conditions	(88)
Picein	Spruce bark	Activate the Nrf2/HO-1/GPX4 axis to enhance osteogenic differentiation of BMSCs	Promote bone regeneration in osteoporotic rats and reduce inflammation levels	(108)
Quercetin	Parasitic loranthus, hawthorn	Activation of the Nrf2/HO-1 signaling pathway and alleviation of oxidative stress damage induced by iron overload	Reduction of osteoporosis induced by iron overload in mouse models	(109)
Aconitine	Aconiti lateralis radix praeparata	Inhibit the phosphorylation of I-κB and p65 in the NF-κB signaling pathway to suppress GPX4 and upregulate ACSL4	Attenuating osteoclast-mediated bone resorption and ferroptosis, improving osteoporosis	(110)
Icariin	Epimedium	Reduce the expression of ferroportin, and increase the expression of osteoblast markers and GPX4 protein	Promote the proliferation and differentiation of osteoblasts to achieve the goal of treating osteoporosis	(111)
Baicalin	Scutellaria baicalensis	Regulate the expression of RANKL and OPG	Hindered the expression of genes related to bone formation and bone resorption in osteoporotic zebrafish	(112)
Aucubin	Eucommia	Inhibit the generation of ROS and intracellular ferrous ions, reduce the activity of MDA, and promote the activity of superoxide dismutase (SOD) and GPX	Inhibit ROS generation and oxidative stress induced and protected against ferroptosis in BMSCs	(113)
Forsythiaside A	Forsythia suspensa fruit	Promote nuclear translocation of Nrf2 and enhance the expression of GPX4	Counteracts the suppression of osteogenic differentiation and mineralization caused by high glucose and high fat (HGHF) in BMSCs	(114)
Zingerone	Dried ginger	Increased ferroptosis sensitivity by p53-mediated regulation of SAT1 and GPX4 expression	Promote ferroptosis of osteoclasts and inhibit the formation of osteoclasts	(115)
Salidroside	Rhodiola	Activate the PI3K/AKT/mTOR signaling pathway and upregulate the protein and gene expression levels of SLC7A11, GPX4, and iron death suppressor protein 1 (FSP1)	Promote the proliferation and osteogenic differentiation of BMSCs in senile osteoporosis, increase the formation of bone trabeculae, and improved the bone microstructure	(116)

### 3.4 Other natural active ingredients

Many natural active substances have a positive effect on the treatment of osteoporosis. The natural compound, named poliumoside, can inhibit the bone degradation and ferroptosis caused by high glucose and high fat (HGHF). The mechanism is to promote the GSH levels and reduce MDA, lipid peroxidation, and mitochondrial reactive oxygen ROS (91). The natural active component, arecoline, can alleviate the inhibitory effect of ferroptosis on the osteogenic process of fish larvae, manifested by increased bone mineralization and upregulation of osteogenic genes. Moreover, it is indicated that heme oxygenase-1 (HO-1) is the key mediator for its inhibition of ferroptosis and promotion of osteogenesis (92). Gnetol l (GT) is a naturally occurring divinyl compound. In the treatment of osteoporosis, it inhibits the differentiation and activity of osteoclasts by promoting lipid peroxidation, depleting intracellular GSH (38). Silymarin is a flavonoid compound extracted from the seeds of milk thistle, and it exhibits significant antioxidant properties. Silymarin has been proven to enhance the expression of RUNX2 and SIRT1, inhibit ferroptosis of osteoblasts, thereby promoting the activity and differentiation of osteoblasts. Moreover, in animal models of osteoporosis, it was found that silymarin can improve bone loss by inhibiting iron depletion (93). Equally important, there are also some natural active ingredients, for instance, geniposide extracted from gardenia flowers, which has demonstrated the effect of inhibiting ferroptosis in other diseases (94), and at the same time can regulate osteoblast apoptosis through the Nrf2/ NF-kB pathway, preventing and treating osteoporosis. This also provides new ideas and strategies for the possible treatment of osteoporosis by geniposide through inhibiting the ferroptosis pathway (95). Furthermore, PAC, which is widely present in plants such as grapes and sea buckthorn, has recently been recognized as having certain potential for treating osteoporosis. Studies have shown that PAC has certain potential in alleviating osteoporosis through the SIRT6/Nrf2/GPX4 pathway. Its manifestation is to improve the structure of bone trabeculae and enhance the expression of key osteogenic proteins (96). In conclusion, natural active substances have the advantages of multi-target and easy acceptance in the treatment of osteoporosis, but also have the disadvantages of complex composition and difficult quality control. There is still a long way to go to use natural active substances to treat human osteoporosis.

### 3.5 Hormones and novel regulatory factors

The suppression of interferon regulatory factor 9 (IRF9) enhances the differentiation of osteoclasts, consequently hindering ferroptosis and potentially offering a new avenue for the treatment of osteoporosis (97). Melatonin prevents ferroptosis in osteoblasts through the activation of the Nrf2/HO-1 signaling pathway, which subsequently enhances bone microstructure both *in vivo* and *in vitro* (23). Moreover, it significantly alleviates glucocorticoid-induced ferroptosis of BMSCs and prevents the occurrence of SIOP by inhibiting ferroptosis through activation of the PI3K/AKT/mTOR signaling pathway (46). The latest research reports that melatonin is a powerful endogenous antioxidant. It can alleviate oxidative stress caused by sodium sulfite (a food additive), inhibit ferroptosis, restore the function of osteoblasts, and reduce bone loss in mice. This study suggests that melatonin is a promising therapeutic agent that can be used for the prevention and treatment of this condition (98). Interestingly, there are also some studies that have focused on the impact of ferroptosis on endothelial cells in osteoporosis. For instance, a recent report indicates that Eldecalcitol (1alpha,25-dihydroxy-2beta-(3-hydroxypropoxy) vitamin D3) may treat osteoporosis by alleviating ferroptosis in endothelial cells. This also provides a new perspective for treating osteoporosis through the ferroptosis pathway (99).

### 3.6 Advanced delivery systems targeting ferroptotic hubs

Although the research on natural ingredients combined with advanced delivery systems is limited, it is still an innovation and a future research direction. Some studies have synthesized iron-suppressing nanoparticles capable of delivering the natural compound curcumin to the bone marrow using tetrahedral framework nucleic acids (tFNA). Experiments conducted both *in vitro* and *in vivo* have demonstrated that nanoparticles can hinder ferroptosis and enhance the osteogenic differentiation of BMSCs by diminishing the overproduction of ROS, decreasing the levels of Fe^2+^, and stimulating the KEAP1/NRF2 pathway, which in turn boosts the expression of GPX4 within the diabetic microenvironment (100). Another study integrated genetic engineering with bone-targeting peptide modification to develop an innovative exosome for bone-targeting engineering. The team synthesized F6-(DSS)6-exo, which effectively delivered curcumin to target specific sites. By inhibiting ferroptosis and ROS, this approach restored the osteogenic differentiation potential of BMSCs and alleviated bone loss in smoking-related osteoporosis (SROP) mouse models (117).

Although few studies have demonstrated the molecular mechanism that directly regulates the relationship between ferroptosis and osteoporosis through the ferroptosis pathway. But in the latest research, it seems that we have seen relatively clear results. The study combines natural derivatives with advanced delivery systems and reports an injectable natural tremella-derived hydrogel that is used to reverse the microenvironment imbalance of osteoporosis mediated by ferroptosis and promote bone regeneration. It also directly confirms that regulating the ferroptosis pathway (improving the endogenous iron metabolism and anti-lipid peroxidation metabolism of osteoblasts) can protect the mitochondrial structure of osteoblasts damaged by ferroptosis (101).

In conclusion, although current research integrating natural active ingredients with advanced delivery systems remains limited, but the natural component delivery system presents a novel approach for treating osteoporosis by precisely modulating the ferroptosis pathway. Future efforts should prioritizeclinical translation and the integration of interdisciplinary technologies to tackle challenges such as delivery efficiency, safety, and individualized treatment, ultimately facilitating the transition from laboratory research toclinical practice.

## 4 Conclusion and outlook

This article reviews the molecular mechanisms of ferroptosis-induced osteoporosis, summarizes the signaling pathways through which ferroptosis affects bone metabolism, and focuses on the three-dimensional regulatory network of iron metabolism disorder, lipid peroxidation and bone homeostasis imbalance. What is more innovative is that from the perspectives of natural product chemistry and molecular pharmacology, we evaluated the potential of natural active ingredients, traditional Chinese medicines and their corresponding targets to inhibit ferroptosis, and also envisioned their combination with advanced delivery systems, highlighting their potential in developing new therapeutic strategies.

However, there remains significant scope for exploration and several issues that require attention in the research on ferroptosis and bone metabolism: currently, research on the regulatory role of ferroptosis in osteoporosis predominantly focuses on two mechanisms: iron overload and lipid peroxidation. We propose the development of next-generation iron chelators specifically targeting bone or the regulation of the hepcidin pathway to mitigate iron overload, thereby alleviating osteoporosis associated with ferroptosis and providing novel strategies for treating iron metabolism-related diseases. Furthermore, as new pathways of ferroptosis are continuously being discovered, the potential for other regulatory mechanisms and triggering factors urgently warrants further exploration.

Research on ferroptosis in senile osteoporosis is limited. Given the aging population, it is essential to establish a scientific animal model to elucidate the relationship between ferroptosis and primary osteoporosis, distinguishing it from other osteoporosis types. Furthermore, the heterogeneity of the disease is a critical factor that warrants consideration. Osteoporosis, a prevalent bone disease, not only encompasses multiple types but also exhibits significant variability in pathogenesis among patients within the same type. This variability suggests that the role of ferroptosis in osteoporosis may differ among individuals, necessitating the development of personalized treatment strategies tailored to each patient’s specific condition. Such personalized approaches can better address patient needs and enhance treatment efficacy.

A notable focus in current research is the precise targeting of key molecules, including GPX4, System Xc-, and FSP1. Treatment strategies encompass gene therapy, the development of small molecule activators or mimics, and methods to enhance cysteine uptake. However, challenges remain in addressing issues related to targeting, safety, and delivery efficiency. For instance, ferroptosis, an emerging form of cell death, is influenced by the systemic regulation of related genes, such as GPX4, which can affect multiple organ systems, particularly vital organs like the liver and heart. These organs exhibit high sensitivity to ferroptosis, and systemic regulation may lead to functional disorders. Therefore, when conducting ferroptosis-related gene therapy, it is crucial to consider the systemic effects and implement corresponding measures to protect these key organs, presenting a significant challenge for the clinical applicability of this approach.

Research is currently underway to prevent and treat osteoporosis utilizing natural active components through the ferroptosis mechanism. These natural active components can inhibit ferroptosis via multiple targets and pathways, thereby offering promising strategies for osteoporosis prevention and treatment. While some positive outcomes have been observed in cell and animal studies, substantial barriers remain before these results can be implemented in clinical settings. First, at the current stage, the relevant clinical studies are mostly divided into two categories: correlation studies and retrospective studies. The former reported that there were differences in the levels of ferroptosis-related indicators between non-osteoporotic subjects and osteoporotic patients (102, 103). The latter indicates that traditional Chinese medicine has also been applied in clinical research. For instance, a retrospective study suggests that Eucommia ulmoides may treat osteoporosis by regulating ferroptosis-related indicators (104). However, the above-mentioned clinical studies have limitations such as small sample size, single research center, and single clinical type of osteoporosis, which lack persuasiveness. That is to say, although conclusive evidence from human clinical or epidemiological studies is still being accumulated, the content related to ferroptosis has not yet become a routine standard for clinical diagnosis. Currently, there is almost no evidence suggesting a direct association between ferroptosis and human osteoporosis. Second, regarding the detection value and reliability of iron death-related targets in the transition from animal models to human clinical applications, we still need to conduct an analysis with caution and optimism. In the relevant clinical studies, most of the indicators measured in human blood are taken, but there is a drawback of low specificity, making it difficult to prove that the changes in these indicators are derived from iron death in the bones. And the key proteins discovered from animal models, such as GPX4, SLC7A11, ACSL4, etc., are extremely difficult and unethical to obtain from healthy human bodies, especially in early bone tissue samples of osteoporosis.

Furthermore, the assessment indicators for osteoporosis in animals and *in vitro* studies are diverse. How these indicators can be effectively linked to the clinical observation parameters of human osteoporosis is one of the key points for achieving clinical translation. Currently, the main assessment indicators for human osteoporosis are based on DXA measurements of bone density values. While the assessment indicators or methods in animal models, such as Micro-CT, directly simulate the degeneration of bone structure in human osteoporosis, and the Micro-CT indicators are highly correlated with the bone mineral density measured by DXA, also indicating the risk of fractures. Additionally, bone turnover biochemical markers (BTMs) reflect the overall rate of bone turnover and correspond to clinical-detected BTMs, which are of great significance for non-invasive detection of disease status and therapeutic efficacy. The evaluation indicators in *in vitro* studies focus on the functions of osteoblasts and osteoclasts, allowing us to better understand the pathological mechanism of osteoporosis and providing a mechanistic perspective for understanding the imbalance in bone turnover.

To translate basic research findings into effective clinical treatments, it is imperative to conduct multi-center, large-sample, high-quality prospective clinical studies to verify the safety and efficacy of these therapeutic strategies. Concurrently, there is a need for the development of more precise detection methods to accurately assess patients’ conditions in clinical practice and to monitor and follow up on treatment effects. Moreover, challenges related to the stability, bioavailability, and complexity of the action mechanisms of these active components significantly hinder their clinical application and warrant further investigation.

In summary, future research will focus on several key areas: First, we will actively explore and identify new therapeutic targets to provide more effective intervention strategies for disease treatment. Second, we will carefully formulate and optimize combination treatment strategies that leverage the synergy of multiple therapeutic methods to enhance their efficacy. Third, we aim to achieve significant breakthroughs in interdisciplinary technologies by integrating advanced methodologies from various fields, including biology, medicine, and information science, which will offer robust technical support for disease research and treatment. Finally, we will vigorously promote translational clinical research to accelerate the conversion of fundamental research findings into clinical applications, ensuring that these results are swiftly translated into actual diagnostic and therapeutic practices to benefit patients.

## References

[1] Subarajan P Arceo-Mendoza RM Camacho PM. Postmenopausal osteoporosis: a review of latest guidelines. Endocrinol Metab Clin N Am. (2024) 53:497–512. doi: 10.1016/j.ecl.2024.08.008, 39448132

[2] Zou Z Liu W Cao L Liu Y He T Peng S . Advances in the occurrence and biotherapy of osteoporosis. Biochem Soc Trans. (2020) 48:1623–36. doi: 10.1042/BST20200005, 32627832

[3] Wright NC Looker AC Saag KG Curtis JR Delzell ES Randall S . The recent prevalence of osteoporosis and low bone mass in the United States based on bone mineral density at the femoral neck or lumbar spine. J Bone Miner Res. (2014) 29:2520–6. doi: 10.1002/jbmr.2269, 24771492 PMC4757905

[4] Osteoporosis and Bone Mineral Diseases Branch of the Chinese Medical Association. Epidemiological investigation of osteoporosis in China and results of special action on “healthy bones”. Chin J Osteoporos Bone Miner Res. (2019) 12:317–8. doi: 10.3969/j.issn.1674-2591.2019.04.001

[5] Hernlund E Svedbom A Ivergård M Compston J Cooper C Stenmark J . Osteoporosis in the European Union: medical management, epidemiology and economic burden. A report prepared in collaboration with the International Osteoporosis Foundation (IOF) and the European Federation of Pharmaceutical Industry Associations (EFPIA). Arch Osteoporos. (2013) 8:136. doi: 10.1007/s11657-013-0136-1, 24113837 PMC3880487

[6] Bedoui S Herold MJ Strasser A. Emerging connectivity of programmed cell death pathways and its physiological implications. Nat Rev Mol Cell Biol. (2020) 21:678–95. doi: 10.1038/s41580-020-0270-8, 32873928

[7] Ursini F Maiorino M. Lipid peroxidation and ferroptosis: the role of GSH and GPx4. Free Radic Biol Med. (2020) 152:175–85. doi: 10.1016/j.freeradbiomed.2020.02.027, 32165281

[8] Dixon SJ Lemberg KM Lamprecht MR Skouta R Zaitsev EM Gleason CE . Ferroptosis: an iron-dependent form of nonapoptotic cell death. Cell. (2012) 149:1060–72. doi: 10.1016/j.cell.2012.03.042, 22632970 PMC3367386

[9] Kim JM Lin C Stavre Z Greenblatt MB Shim JH. Osteoblast-osteoclast communication and bone homeostasis. Cells. (2020) 9:2073. doi: 10.3390/cells9092073, 32927921 PMC7564526

[10] Yang J Li Q Feng Y Zeng Y. Iron deficiency and iron deficiency anemia: potential risk factors in bone loss. Int J Mol Sci. (2023) 24:6891. doi: 10.3390/ijms24086891, 37108056 PMC10138976

[11] Zhao GY Zhao LP He YF Li GF Gao C Li K . A comparison of the biological activities of human osteoblast hFOB1.19 between iron excess and iron deficiency. Biol Trace Elem Res. (2012) 150:487–95. doi: 10.1007/s12011-012-9511-9, 23054865

[12] Baschant U Rauner M Balaian E Weidner H Roetto A Platzbecker U . Wnt5a is a key target for the pro-osteogenic effects of iron chelation on osteoblast progenitors. Haematologica. (2016) 101:1499–507. doi: 10.3324/haematol.2016.144808, 27540134 PMC5479606

[13] Chen BY Pathak JL Lin HY Guo WQ Chen WJ Luo G . Inflammation triggers chondrocyte ferroptosis in TMJOA via HIF-1α/TFRC. J Dent Res. (2024) 103:712–22. doi: 10.1177/00220345241242389, 38766865

[14] Stockwell BR Friedmann Angeli JP Bayir H Bush AI Conrad M Dixon SJ . Ferroptosis: a regulated cell death Nexus linking metabolism, redox biology, and disease. Cell. (2017) 171:273–85. doi: 10.1016/j.cell.2017.09.021, 28985560 PMC5685180

[15] Feng Y He PY Kong WD Cen WJ Wang PL Liu C . Apoptosis-promoting properties of miR-3074-5p in MC3T3-E1 cells under iron overload conditions. Cell Mol Biol Lett. (2021) 26:37. doi: 10.1186/s11658-021-00281-w, 34399682 PMC8365891

[16] Zhang Y Zhai W Zhao M Li D Chai X Cao X . Effects of iron overload on the bone marrow microenvironment in mice. PLoS One. (2015) 10:e0120219. doi: 10.1371/journal.pone.0120219, 25774923 PMC4361683

[17] Zhang H Wang A Shen G Wang X Liu G Yang F . Hepcidin-induced reduction in iron content and PGC-1β expression negatively regulates osteoclast differentiation to play a protective role in postmenopausal osteoporosis. Aging. (2021) 13:11296–314. doi: 10.18632/aging.202817, 33820875 PMC8109081

[18] Yang J Dong D Luo X Zhou J Shang P Zhang H. Iron overload-induced osteocyte apoptosis stimulates osteoclast differentiation through increasing osteocytic RANKL production *in vitro*. Calcif Tissue Int. (2020) 107:499–509. doi: 10.1007/s00223-020-00735-x, 32995951

[19] Shah R Shchepinov MS Pratt DA. Resolving the role of lipoxygenases in the initiation and execution of ferroptosis. ACS Cent Sci. (2018) 4:387–96. doi: 10.1021/acscentsci.7b00589, 29632885 PMC5879472

[20] Gaschler MM Stockwell BR. Lipid peroxidation in cell death. Biochem Biophys Res Commun. (2017) 482:419–25. doi: 10.1016/j.bbrc.2016.10.086, 28212725 PMC5319403

[21] Liang D Minikes AM Jiang X. Ferroptosis at the intersection of lipid metabolism and cellular signaling. Mol Cell. (2022) 82:2215–27. doi: 10.1016/j.molcel.2022.03.022, 35390277 PMC9233073

[22] Zhang Y Huang X Qi B Sun C Sun K Liu N . Ferroptosis and musculoskeletal diseases: “Iron maiden” cell death may be a promising therapeutic target. Front Immunol. (2022) 13:972753. doi: 10.3389/fimmu.2022.972753, 36304454 PMC9595130

[23] Ma H Wang X Zhang W Li H Zhao W Sun J . Melatonin suppresses ferroptosis induced by high glucose via activation of the Nrf2/HO-1 signaling pathway in type 2 diabetic osteoporosis. Oxid Med Cell Longev. (2020) 2020:1–18. doi: 10.1155/2020/9067610, 33343809 PMC7732386

[24] Xu P Lin B Deng X Huang K Zhang Y Wang N. VDR activation attenuates osteoblastic ferroptosis and senescence by stimulating the Nrf2/GPX4 pathway in age-related osteoporosis. Free Radic Biol Med. (2022) 193:720–35. doi: 10.1016/j.freeradbiomed.2022.11.013, 36402439

[25] Liu X Wang T Wang W Liang X Mu Y Xu Y . Emerging potential therapeutic targets of ferroptosis in skeletal diseases. Oxid Med Cell Longev. (2022) 2022:3112388. doi: 10.1155/2022/3112388, 35941905 PMC9356861

[26] Duan JY Lin X Xu F Shan SK Guo B Li FX . Ferroptosis and its potential role in metabolic diseases: a curse or revitalization? Front Cell Dev Biol. (2021) 9:701788. doi: 10.3389/fcell.2021.701788, 34307381 PMC8299754

[27] Zhao Y Du Y Gao Y Xu Z Zhao D Yang M. ATF3 regulates osteogenic function by mediating osteoblast ferroptosis in type 2 diabetic osteoporosis. Dis Markers. (2022) 2022:9872243. doi: 10.1155/2022/9872243, 36340581 PMC9629949

[28] Doll S Freitas FP Shah R Aldrovandi M da Silva MC Ingold I . FSP1 is a glutathione-independent ferroptosis suppressor. Nature. (2019) 575:693–8. doi: 10.1038/s41586-019-1707-0, 31634899

[29] Bersuker K Hendricks JM Li Z Magtanong L Ford B Tang PH . The CoQ oxidoreductase FSP1 acts parallel to GPX4 to inhibit ferroptosis. Nature. (2019) 575:688–92. doi: 10.1038/s41586-019-1705-2, 31634900 PMC6883167

[30] Toxqui L Vaquero MP. Chronic iron deficiency as an emerging risk factor for osteoporosis: a hypothesis. Nutrients. (2015) 7:2324–44. doi: 10.3390/nu7042324, 25849944 PMC4425147

[31] Balogh E Tolnai E Nagy B Jr Nagy B Balla G Balla J . Iron overload inhibits osteogenic commitment and differentiation of mesenchymal stem cells via the induction of ferritin. Biochim Biophys Acta. (2016) 1862:1640–9. doi: 10.1016/j.bbadis.2016.06.003, 27287253

[32] Jiang Z Wang H Qi G Jiang C Chen K Yan Z. Iron overload-induced ferroptosis of osteoblasts inhibits osteogenesis and promotes osteoporosis: an *in vitro* and *in vivo* study. IUBMB Life. (2022) 74:1052–69. doi: 10.1002/iub.2656, 35638167

[33] Ruan B Dong J Wei F Huang Z Yang B Zhang L . DNMT aberration-incurred GPX4 suppression prompts osteoblast ferroptosis and osteoporosis. Bone Res. (2024) 12:68. doi: 10.1038/s41413-024-00365-1, 39617773 PMC11609303

[34] Pan C Wang K Hong R Wang X Zhang Y Fan Z . Chronic microcystin-leucine-arginine exposure induces osteoporosis by breaking the balance of osteoblasts and osteoclasts. Environ Res. (2024) 263:120098. doi: 10.1016/j.envres.2024.120098, 39366441

[35] Xu Z Sun W Li Y Ling S Zhao C Zhong G . The regulation of iron metabolism by hepcidin contributes to unloading-induced bone loss. Bone. (2017) 94:152–61. doi: 10.1016/j.bone.2016.09.023, 27686598

[36] Ni S Yuan Y Qian Z Zhong Z Lv T Kuang Y . Hypoxia inhibits RANKL-induced ferritinophagy and protects osteoclasts from ferroptosis. Free Radic Biol Med. (2021) 169:271–82. doi: 10.1016/j.freeradbiomed.2021.04.027, 33895289

[37] Qu Z Zhang B Kong L Zhang Y Zhao Y Gong Y . Myeloid zinc finger 1 knockdown promotes osteoclastogenesis and bone loss in part by regulating RANKL-induced ferroptosis of osteoclasts through Nrf2/GPX4 signaling pathway. J Leukoc Biol. (2024) 115:946–57. doi: 10.1093/jleuko/qiae011, 38266238

[38] Peng X Zhao W Yang F Wei Y Zhang Y Chen S . Gnetol targeting of TNFAIP3 promotes SLC7A11 ubiquitination and ferroptosis in osteoclasts to ameliorate osteoporosis. Phytomedicine. (2025) 148:157422. doi: 10.1016/j.phymed.2025.157422, 41151451

[39] Cui J Shibata Y Zhu T Zhou J Zhang J. Osteocytes in bone aging: advances, challenges, and future perspectives. Ageing Res Rev. (2022) 77:101608. doi: 10.1016/j.arr.2022.101608 35283289

[40] Yin Y Chen GJ Yang C Wang JJ Peng JF Huang XF . Osteocyte ferroptosis induced by ATF3/TFR1 contributes to cortical bone loss during ageing. Cell Prolif. (2024) 57:e13657. doi: 10.1111/cpr.13657, 38764128 PMC11471391

[41] Shi Y Tang Q Sheng S Jiang H Jin C Zhou C . PSMD14 stabilizes SLC7A11 to ameliorate glucocorticoid-induced osteoporosis by suppressing osteocyte ferroptosis. Adv Sci. (2025) 12:e14902. doi: 10.1002/advs.202414902, 40444470 PMC12376700

[42] Yang Y Lin Y Wang M Yuan K Wang Q Mu P . Targeting ferroptosis suppresses osteocyte glucolipotoxicity and alleviates diabetic osteoporosis. Bone Res. (2022) 10:26. doi: 10.1038/s41413-022-00198-w, 35260560 PMC8904790

[43] Sun F Zhou JL Liu ZL Jiang ZW Peng H. Dexamethasone induces ferroptosis via P53/SLC7A11/GPX4 pathway in glucocorticoid-induced osteonecrosis of the femoral head. Biochem Biophys Res Commun. (2022) 602:149–55. doi: 10.1016/j.bbrc.2022.02.112, 35276555

[44] Lin Y Shen X Ke Y Lan C Chen X Liang B . Activation of osteoblast ferroptosis via the METTL3/ASK1-p38 signaling pathway in high glucose and high fat (HGHF)-induced diabetic bone loss. FASEB J. (2022) 36:e22147. doi: 10.1096/fj.202101610R, 35104016

[45] Liu J Ren Z Yang L Zhu L Li Y Bie C . The NSUN5-FTH1/FTL pathway mediates ferroptosis in bone marrow-derived mesenchymal stem cells. Cell Death Discov. (2022) 8:99. doi: 10.1038/s41420-022-00902-z, 35249107 PMC8898311

[46] Li M Yang N Hao L Zhou W Li L Liu L . Melatonin inhibits the ferroptosis pathway in rat bone marrow mesenchymal stem cells by activating the PI3K/AKT/mTOR signaling axis to attenuate steroid-induced osteoporosis. Oxid Med Cell Longev. (2022) 2022:8223737. doi: 10.1155/2022/8223737, 36035224 PMC9410838

[47] Ono T Hayashi M Sasaki F Nakashima T. RANKL biology: bone metabolism, the immune system, and beyond. Inflamm Regen. (2020) 40:2. doi: 10.1186/s41232-019-0111-3, 32047573 PMC7006158

[48] Hou JM Xue Y Lin QM. Bovine lactoferrin improves bone mass and microstructure in ovariectomized rats via OPG/RANKL/RANK pathway. Acta Pharmacol Sin. (2012) 33:1277–84. doi: 10.1038/aps.2012.83, 22902986 PMC4002710

[49] Jia P Xu YJ Zhang ZL Li K Li B Zhang W . Ferric ion could facilitate osteoclast differentiation and bone resorption through the production of reactive oxygen species. J Orthop Res. (2012) 30:1843–52. doi: 10.1002/jor.22133, 22570238

[50] Lao Z Chen X Chen X Zhang H Zhang Z Bian Y . Vertebral osteoporosis in systemic lupus erythematosus: a possible involvement of inflammation-related osteoblast ferroptosis. J Inflamm Res. (2025) 18:5587–99. doi: 10.2147/JIR.S523051, 40303004 PMC12039848

[51] Jin C Tan K Yao Z Lin BH Zhang DP Chen WK . A novel anti-osteoporosis mechanism of VK2: interfering with ferroptosis via AMPK/SIRT1 pathway in type 2 diabetic osteoporosis. J Agric Food Chem. (2023) 71:2745–61. doi: 10.1021/acs.jafc.2c05632, 36719855

[52] Huang B Jiang J Ou X Hao M Shao H. ELAVL1 promotes ferroptosis-induced inhibition of osteogenic differentiation in diabetic osteoporosis by downregulating SIRT1. Tissue Cell. 97. doi: 10.1016/j.tice.2025.103060, 40753732

[53] Dong Y Kang H Peng R Liu Z Liao F Hu SA . A clinical-stage Nrf2 activator suppresses osteoclast differentiation via the iron-ornithine axis. Cell Metab. (2024) 36:1679–1695.e6. doi: 10.1016/j.cmet.2024.03.005, 38569557

[54] Bao J Yan Y Zuo D Zhuo Z Sun T Lin H . Iron metabolism and ferroptosis in diabetic bone loss: from mechanism to therapy. Front Nutr. (2023) 10:1178573. doi: 10.3389/fnut.2023.1178573, 37215218 PMC10196368

[55] Wu X Fang X Lu F Chen Q Liu J Zheng L. An update on the role of ferroptosis in the pathogenesis of osteoporosis. EFORT Open Rev. (2024) 9:712–22. doi: 10.1530/EOR-23-0148, 39087516 PMC11370720

[56] Almeida M Laurent MR Dubois V Claessens F O'Brien CA Bouillon R . Estrogens and androgens in skeletal physiology and pathophysiology. Physiol Rev. (2017) 97:135–87. doi: 10.1152/physrev.00033.2015, 27807202 PMC5539371

[57] Jiang X Kagan R. Hormone therapy for postmenopausal osteoporosis management. Climacteric. (2022) 25:50–5. doi: 10.1080/13697137.2021.1957818, 34402365

[58] Ferron M Wei J Yoshizawa T Del Fattore A DePinho RA Teti A . Insulin signaling in osteoblasts integrates bone remodeling and energy metabolism. Cell. (2010) 142:296–308. doi: 10.1016/j.cell.2010.06.003, 20655470 PMC2910411

[59] Srivastava M Deal C. Osteoporosis in elderly: prevention and treatment. Clin Geriatr Med. (2002) 18:529–55. doi: 10.1016/s0749-0690(02)00022-8 12424871

[60] Ru Q Li Y Xie W Ding Y Chen L Xu G . Fighting age-related orthopedic diseases: focusing on ferroptosis. Bone Res. (2023) 11:12. doi: 10.1038/s41413-023-00247-y, 36854703 PMC9975200

[61] Wu D Wen X Liu W Hu H Ye B Zhou Y. Comparison of the effects of deferasirox, deferoxamine, and combination of deferasirox and deferoxamine on an aplastic anemia mouse model complicated with iron overload. Drug Des Devel Ther. (2018) 12:1081–91. doi: 10.2147/DDDT.S161086, 29760547 PMC5937503

[62] Zheng N Lu J Sheng H. Feasibility and safety of deferoxamine mesylate in the treatment of postmenopausal osteoporosis. Chin J Osteoporos. (2019) 25:998–1001. doi: 10.3969/j.issn.1006-7108

[63] Zhang J Hu W Ding C Yao G Zhao H Wu S. Deferoxamine inhibits iron-uptake stimulated osteoclast differentiation by suppressing electron transport chain and MAPKs signaling. Toxicol Lett. (2019) 313:50–9. doi: 10.1016/j.toxlet.2019.06.007, 31238089

[64] Kang H Yan Y Jia P Yang K Guo C Chen H . Desferrioxamine reduces ultrahigh-molecular-weight polyethylene-induced osteolysis by restraining inflammatory osteoclastogenesis via heme oxygenase-1. Cell Death Dis. (2016) 7:e2435. doi: 10.1038/cddis.2016.339, 27787522 PMC5133998

[65] Guo C Yang K Yan Y Yan D Cheng Y Yan X . SF-deferoxamine, a bone-seeking angiogenic drug, prevents bone loss in estrogen-deficient mice. Bone. (2019) 120:156–65. doi: 10.1016/j.bone.2018.10.025, 30385424

[66] Chen B Yan YL Liu C Bo L Li GF Wang H . Therapeutic effect of deferoxamine on iron overload-induced inhibition of osteogenesis in a zebrafish model. Calcif Tissue Int. (2014) 94:353–60. doi: 10.1007/s00223-013-9817-4, 24414856

[67] González Suárez I Fernández Martín JL Naves Díaz M Cannata Andía JB. Effect of desferrioxamine and deferiprone on osteocalcin secretion in osteoblast-type cells. Nefrologia. (2003) 23:27–31. 12778850

[68] Luo C Xu W Tang X Liu X Cheng Y Wu Y . Canonical Wnt signaling works downstream of iron overload to prevent ferroptosis from damaging osteoblast differentiation. Free Radic Biol Med. (2022) 188:337–50. doi: 10.1016/j.freeradbiomed.2022.06.236, 35752374

[69] Messa E Carturan S Maffè C Pautasso M Bracco E Roetto A . Deferasirox is a powerful NF- B inhibitor in myelodysplastic cells and in leukemia cell lines acting independently from cell iron deprivation by chelation and reactive oxygen species scavenging. Haematologica. (2010) 95:1308–16. doi: 10.3324/haematol.2009.016824, 20534700 PMC2913079

[70] Zhao G Cheng Q Wang B Di D. Effects of deferiprone on biologic activity of murine preosteoblasts of MC3T3-E1. Chin J Osteoporos Bone Miner Res. (2017):121–4. doi: 10.3969/j.issn.1674-2591.2017.02.005

[71] Casale M Citarella S Filosa A de Michele E Palmieri F Ragozzino A . Endocrine function and bone disease during long-term chelation therapy with deferasirox in patients with β-thalassemia major. Am J Hematol. (2014) 89:1102–6. doi: 10.1002/ajh.23844, 25197009

[72] Chen B Wang S Wang X Ye J Liu L Gao Y . Effects of deferasirox on bone mass and construction in ovariectomy mice with iron accumulation. Chin J Osteoporos Bone Miner Res. (2017) 10:140–5. doi: 10.3969/j.issn.1674-2591.2017.02.008

[73] Zhao G-y Cheng Q Wang B Wang L Zhang P Xu Y. Deferasirox inhibit differentiation of mouse RAW264.7 monocytes into osteoclasts through NF-κB signal. Chin J Osteoporos Bone Miner Res. (2016) 9:149–56. doi: 10.3969/j.issn.1674-2591.2016.02.008

[74] Qu X Sun Z Wang Y Ong HS. Zoledronic acid promotes osteoclasts ferroptosis by inhibiting FBXO9-mediated p53 ubiquitination and degradation. PeerJ. (2021) 9:e12510. doi: 10.7717/peerj.12510, 35003915 PMC8684721

[75] Cai Y Jun G Zhuang X. Metformin treatment reduces the incidence of osteoporosis: a two-sample Mendelian randomized study. Osteoporos Int. (2024) 35:1089–98. doi: 10.1007/s00198-023-07013-0, 38536446 PMC11136748

[76] Liu Y Fu Z Wang X Yang Q Liu S Zhu D. Metformin attenuates diabetic osteoporosis by suppressing ferroptosis via the AMPK/Nrf2 pathway. Front Pharmacol. (2025) 16:1527316. doi: 10.3389/fphar.2025.1527316, 40206070 PMC11979264

[77] Cao S Wei Y Yue Y Chen Y Qian J Wang D . Rosiglitazone retards the progression of iron overload-induced osteoarthritis by impeding chondrocyte ferroptosis. iScience. (2024) 27:110526. doi: 10.1016/j.isci.2024.110526, 39224514 PMC11366908

[78] Li P Wang Y Yan Q Yang Y Zhu R Ma J . Fructus *Ligustri Lucidi* inhibits ferroptosis in ovariectomy-induced osteoporosis in rats via the Nrf2/HO-1 signaling pathway. Biomed Rep. (2023) 20:27. doi: 10.3892/br.2023.1715, 38259585 PMC10801352

[79] Hao J Bei J Li Z Han M Ma B Ma P . Qing’e pill inhibits osteoblast ferroptosis via ATM serine/threonine kinase (ATM) and the PI3K/AKT pathway in primary osteoporosis. Front Pharmacol. (2022) 13:902102. doi: 10.3389/fphar.2022.902102, 35865965 PMC9294279

[80] Li X Hu W Gan F Ye B Wu D Jiang X. Protective effect and mechanism of Duzhong-Xuduan medicinal pair on ovariectomized osteoporosis rats by regulating ferroptosis pathway. Chin Arch Tradit Chin Med. (2023) 41:103–6. doi: 10.13193/j.issn.1673-7717.2023.09.021

[81] Hu L Luo M Zhu X Wang Z Yan C Wu L . Psoraleae Fructus combined with walnut kernels improves postmenopausal osteoporosis by inhibiting ferroptosis through the Nrf2/GPX4/SLC7A11 pathway. Phytomedicine. (2025) 148:157319. doi: 10.1016/j.phymed.2025.157319, 41016294

[82] Ma Y Hu J Song C Li P Cheng Y Wang Y . Er-Xian decoction attenuates ovariectomy-induced osteoporosis by modulating fatty acid metabolism and IGF1/PI3K/AKT signaling pathway. J Ethnopharmacol. (2023) 301:115835. doi: 10.1016/j.jep.2022.115835 36252878

[83] Zhang Y Fang S Li Q Sun C Liu N Sun K . Effects of Bugu Shengsui decoction on related indicators of oxidative stress and Ferroptosis in rats with osteoporosis. Chin J Inf TCM. (2022) 29:75–9. doi: 10.19879/j.cnki.1005-5304.202109060

[84] Piwowar A Rembiałkowska N Rorbach-Dolata A Garbiec A Ślusarczyk S Dobosz A . Anemarrhenae asphodeloides rhizoma extract enriched in mangiferin protects PC12 cells against a neurotoxic agent-3-nitropropionic acid. Int J Mol Sci. (2020) 21:2510. doi: 10.3390/ijms21072510, 32260390 PMC7177269

[85] Szandruk M Merwid-Ląd A Szeląg A. The impact of mangiferin from *Belamcanda chinensis* on experimental colitis in rats. Inflammopharmacology. (2018) 26:571–81. doi: 10.1007/s10787-017-0337-0, 28337639 PMC5859701

[86] Popović Z Krstić-Milošević D Marković M Vidaković V Bojović S. *Gentiana asclepiadea* L. from two high mountainous habitats: inter- and intrapopulation variability based on species' phytochemistry. Plants. (2021) 10:140. doi: 10.3390/plants10010140, 33445468 PMC7827789

[87] Deng X Lin B Wang F Xu P Wang N. Mangiferin attenuates osteoporosis by inhibiting osteoblastic ferroptosis through Keap1/Nrf2/SLC7A11/GPX4 pathway. Phytomedicine. (2024) 124:155282. doi: 10.1016/j.phymed.2023.155282, 38176266

[88] Wei F Ruan B Dong J Yang B Zhang G Kelvin Yeung WK . Asperosaponin VI inhibition of DNMT alleviates GPX4 suppression-mediated osteoblast ferroptosis and diabetic osteoporosis. J Adv Res. (2025) 75:331–44. doi: 10.1016/j.jare.2024.11.036 39647633 PMC12536592

[89] Li Y Li F. Mechanism and prospect of gastrodin in osteoporosis, bone regeneration, and osseointegration. Pharmaceuticals. (2022) 15:1432. doi: 10.3390/ph15111432, 36422561 PMC9698149

[90] Zhong G Chen J Li Y Han Y Wang M Nie Q . Ginsenoside Rg3 attenuates myocardial ischemia/reperfusion-induced ferroptosis via the keap1/Nrf2/GPX4 signaling pathway. BMC Complement Med Ther. (2024) 24:247. doi: 10.1186/s12906-024-04492-4, 38926825 PMC11209975

[91] Xu CY Xu C Xu YN Du SQ Dai ZH Jin SQ . Poliumoside protects against type 2 diabetes-related osteoporosis by suppressing ferroptosis via activation of the Nrf2/GPX4 pathway. Phytomedicine. (2024) 125:155342. doi: 10.1016/j.phymed.2024.155342, 38295665

[92] Jiang Z Deng L Xiang G Xu X Wang Y. A mechanistic study of the osteogenic effect of arecoline in an osteoporosis model: inhibition of iron overload-induced osteogenesis by promoting heme oxygenase-1 expression. Antioxidants. (2024) 13:430. doi: 10.3390/antiox13040430, 38671878 PMC11047558

[93] Tao ZS Li TL Wei S. Silymarin prevents iron overload induced bone loss by inhibiting oxidative stress in an ovariectomized animal model. Chem Biol Interact. (2022) 366:110168. doi: 10.1016/j.cbi.2022.110168, 36087815

[94] Shen Y Wang X Shen X Wang Y Wang S Zhang Y . Geniposide possesses the protective effect on myocardial injury by inhibiting oxidative stress and ferroptosis via activation of the Grsf1/GPx4 Axis. Front Pharmacol. (2022) 13:879870. doi: 10.3389/fphar.2022.879870, 35600863 PMC9117627

[95] Xiao Y Zhang S Ye Y Chen J Xu Y. Geniposide suppressed OX-LDL-induced osteoblast apoptosis by regulating the NRF2/NF-κB signaling pathway. J Orthop Surg Res. (2023) 18:641. doi: 10.1186/s13018-023-04125-5, 37649066 PMC10466864

[96] Ma RX Lin BH Feng SX Bu YT Chen ZH Huang YX . Evaluation of proanthocyanidins in treating type 2 diabetic osteoporosis via SIRT6/Nrf2/GPX4 pathways. FASEB J. (2025) 39:e70487. doi: 10.1096/fj.202403032R, 40178920

[97] Lan C Zhou X Shen X Lin Y Chen X Lin J . Suppression of IRF9 promotes osteoclast differentiation by decreased ferroptosis via STAT3 activation. Inflammation. (2024) 47:99–113. doi: 10.1007/s10753-023-01896-1, 37804406

[98] He Q Xie L Peng H Xiao X Yu T. Melatonin alleviates sodium sulfite-induced osteoporosis in mice via suppression of the ferroptosis pathway. Apoptosis. (2025) 30:2301–15. doi: 10.1007/s10495-025-02135-8, 40681799 PMC12474642

[99] Dai Y Jiang Y Li X Sun J Liu H Liu B . Eldecalcitol ameliorates type 2 diabetic osteoporosis by attenuating endothelial ferroptosis via the SOCE/O-GlcNAcylation axis. Free Radic Biol Med. (2025) 241:447–58. doi: 10.1016/j.freeradbiomed.2025.09.041, 41005739

[100] Li Y Cai Z Ma W Bai L Luo E Lin Y. A DNA tetrahedron-based ferroptosis-suppressing nanoparticle: superior delivery of curcumin and alleviation of diabetic osteoporosis. Bone Res. (2024) 12:14. doi: 10.1038/s41413-024-00319-7 38424439 PMC10904802

[101] Ye C Xu J Shi L Zong C Ji W Lu Y . Injectable natural Tremella-derived hydrogel for reversing ferroptosis-mediated osteoporotic microenvironment imbalance and promoting osteoregeneration. Biomaterials. (2026) 324:123532. doi: 10.1016/j.biomaterials.2025.123532, 40660644

[102] Xia Y Ge G Xiao H Wu M Wang T Gu C . REPIN1 regulates iron metabolism and osteoblast apoptosis in osteoporosis. Cell Death Dis. (2023) 14:631. doi: 10.1038/s41419-023-06160-w, 37749079 PMC10519990

[103] Lin S Wang S Liu S Yang B Tang Z Dong Z . Role of ferroptosis-related serological biomarkers in the pathogenesis of postmenopausal osteoporosis and construction of a diagnostic nomogram. Chin J Osteoporos. (2023) 29:625–30. doi: 10.3969/j.issn.1006-7108.2023.05.001

[104] Chen S Cao W Si Z. Based on the SLC7A11/GPX4 theory to explore the clinical efficacy of *Eucommia* decoction in the treatment of postmenopausal osteoporosis. Shenzhen J Integr Tradit Chin Western Med. (2024) 34:5–8. doi: 10.16458/j.cnki.1007-0893.2024.19.002

[105] Jiang Z Qi G He X Yu Y Cao Y Zhang C . Ferroptosis in osteocytes as a target for protection against 711 postmenopausal osteoporosis. Adv Sci. (2024) 11:e2307388. doi: 10.1002/advs.20230738 PMC10966575 38233202

[106] Rossi F Perrotta S Bellini G Luongo L Tortora C Siniscalco D . Iron overload causes osteoporosis in thalassemia major patients through interaction with transient receptor potential vanilloid type 1 (TRPV1) channels. Haematologica. (2014) 99:1876–84. doi: 10.3324/haematol.2014.104463, 25216685 PMC4258755

[107] Lan D Yao C Li X Liu H Wang D Wang Y . Tocopherol attenuates the oxidative stress of BMSCs by inhibiting ferroptosis through the PI3k/AKT/mTOR pathway. Front Bioeng Biotechnol. (2022) 10:938520. doi: 10.3389/fbioe.2022.938520, 36061427 PMC9428255

[108] Huang L Wang J Yu J Bian M Xiang X Han G . Picein alleviates oxidative stress and promotes bone regeneration in osteoporotic bone defect by inhibiting ferroptosis via Nrf2/HO-1/GPX4 pathway. Environ Toxicol. (2024) 39:4066–85. doi: 10.1002/tox.24239, 38727095

[109] Xue C Luo H Wang L Deng Q Kui W Da W . Quercetin protects against iron overload-induced osteoporosis through activating the Nrf2/HO-1 pathway. Life Sci. (2023) 322:121326. doi: 10.1016/j.lfs.2022.121326 36639053

[110] Xue C Luo H Wang L Deng Q Kui W Da W . Aconine attenuates osteoclast-mediated bone resorption and ferroptosis to improve osteoporosis via inhibiting NF-κB signaling. Front Endocrinol. (2023) 14:1234563. doi: 10.3389/fendo.2023.1234563, 38034017 PMC10682992

[111] Fu Y Liu Y Hu X Fu Q Han D Sun G. Effect of icariin on proliferation and differentiation of rat osteoblasts based on ferroptosis pathway. Lishizhen Med Meteria Medica Res. (2022) 33:2100–3. doi: 10.3969/j.issn.1008-0805.2022.09.13

[112] Zhao Y Wang HL Li TT Yang F Tzeng CM. Baicalin ameliorates dexamethasone-induced osteoporosis by regulation of the RANK/RANKL/OPG signaling pathway. Drug Des Devel Ther. (2020) 14:195–206. doi: 10.2147/DDDT.S225516, 32021104 PMC6970258

[113] Zheng Y Sun R Yang H Gu T Han M Yu C . Aucubin promotes BMSCs proliferation and differentiation of postmenopausal osteoporosis patients by regulating ferroptosis and BMP2 signalling. J Cell Mol Med. (2025) 29:e70288. doi: 10.1111/jcmm.70288, 39823248 PMC11740986

[114] Huang YX Bu YT Zhang YK Wang YK Guo YF Jin C . Forsythiaside a suppresses ferroptosis and mitigates type 2 diabetes osteoporosis through the NRF2/GPX4 Axis. Food Sci Nutr. (2025) 13:e70991. doi: 10.1002/fsn3.70991, 41036508 PMC12479373

[115] Li H Cao F Liu D Tao L. Zingerone treats postmenopausal osteoporosis via increased ferroptosis sensitivity by p53-mediated regulation of SAT1 and GPX4 expression. Commun Biol. (2025) 8:1367. doi: 10.1038/s42003-025-08751-z, 41006700 PMC12474940

[116] Li Z Yang S Liu W Ling L Hu H Cao Y . Salidroside alleviates senile osteoporosis in SAMP8 mice by inhibiting the ferroptosis of bone marrow stromal cells via PI3K/AKT/mTOR signaling pathway. Int Immunopharmacol. (2025) 167:115703. doi: 10.1016/j.intimp.2025.115703, 41115354

[117] Wang Y Sun L Dong Z Zhang T Wang L Cao Y . Targeted inhibition of ferroptosis in bone marrow mesenchymal stem cells by engineered exosomes alleviates bone loss in smoking-related osteoporosis. Materials Today Bio. (2025) 31:101501. doi: 10.1016/j.mtbio.2025.101501, 39944529 PMC11815285

